# Updates and Knowledge Gaps in Cholesteatoma Research

**DOI:** 10.1155/2015/854024

**Published:** 2015-03-18

**Authors:** Chin-Lung Kuo, An-Suey Shiao, Matthew Yung, Masafumi Sakagami, Holger Sudhoff, Chih-Hung Wang, Chyong-Hsin Hsu, Chiang-Feng Lien

**Affiliations:** ^1^Department of Otolaryngology-Head and Neck Surgery, Taipei Veterans General Hospital, No. 201, Section 2, Shipai Road, Beitou District, Taipei City 11217, Taiwan; ^2^Institute of Brain Science, National Yang-Ming University, No. 155, Section 2, Linong Street, Taipei City 11221, Taiwan; ^3^Department of Otolaryngology, National Yang-Ming University School of Medicine, No. 155, Section 2, Linong Street, Taipei City 11221, Taiwan; ^4^Department of Otolaryngology, Taoyuan Armed Forces General Hospital, No. 168, Zhongxing Road, Longtan District, Taoyuan City 32551, Taiwan; ^5^Department of Otolaryngology-Head and Neck Surgery, Tri-Service General Hospital, National Defense Medical Center, No. 325, Section 2, Chenggong Road, Neihu District, Taipei City 114, Taiwan; ^6^Department of Otolaryngology, Ipswich Hospital NHS Trust, Heath Road, Ipswich, Suffolk IP4 5PD, UK; ^7^Department of Otolaryngology-Head and Neck Surgery, Hyogo College of Medicine, 1-1 Mukogawacho, Nishinomiya, Hyogo Prefecture 663-8131, Japan; ^8^Department of Otolaryngology and Head and Neck Surgery, Klinikum Bielefeld, Teutoburger Straße 50, 33604 Bielefeld, Germany; ^9^Department of Pediatrics, Mackay Memorial Hospital, No. 92, Section 2, Zhongshan N. Road, Taipei City 10449, Taiwan

## Abstract

The existence of acquired cholesteatoma has been recognized for more than three centuries; however, the nature of the disorder has yet to be determined. Without timely detection and intervention, cholesteatomas can become dangerously large and invade intratemporal structures, resulting in numerous intra- and extracranial complications. Due to its aggressive growth, invasive nature, and the potentially fatal consequences of intracranial complications, acquired cholesteatoma remains a cause of morbidity and death for those who lack access to advanced medical care. Currently, no viable nonsurgical therapies are available. Developing an effective management strategy for this disorder will require a comprehensive understanding of past progress and recent advances. This paper presents a brief review of background issues related to acquired middle ear cholesteatoma and deals with practical considerations regarding the history and etymology of the disorder. We also consider issues related to the classification, epidemiology, histopathology, clinical presentation, and complications of acquired cholesteatoma and examine current diagnosis and management strategies in detail.

## 1. Introduction

Cholesteatoma is a well-demarcated noncancerous cystic lesion derived from an abnormal growth of keratinizing squamous epithelium in the temporal bone [1–3], which is commonly characterized as “skin in the wrong place” [4, 5]. Cholesteatoma results from the enzymatic activity of the cholesteatoma matrix. This abnormal growth is locally invasive and capable of causing the destruction of structures in the middle ear cleft. Furthermore, squamous epithelium may be rendered destructive in an environment of chronic infection, thereby enhancing the osteolytic effects of cholesteatoma [6]. Owing to the fatal capacity of intracranial complications, cholesteatomas remain a cause of pediatric morbidity and death for those who lack access to advanced medical care [7, 8].

**Figure 1:**
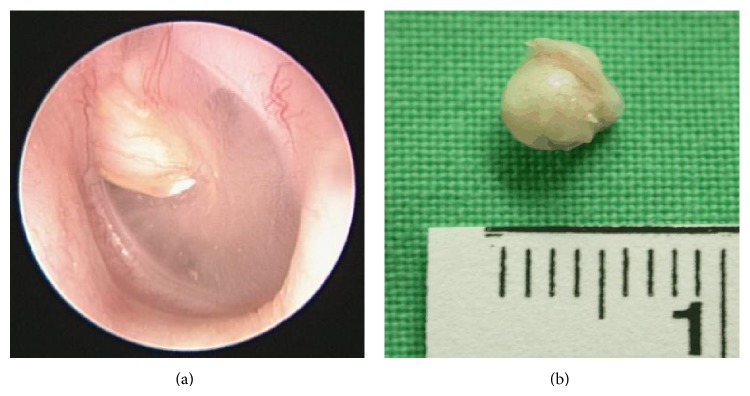
(a) Congenital cholesteatoma, anterosuperior quadrant of eardrum (left ear). A white mass is located behind an intact eardrum without prior otitis media or history of otologic procedures. (b) Dissected round mass with whitish pearly appearance.

**Figure 2:**
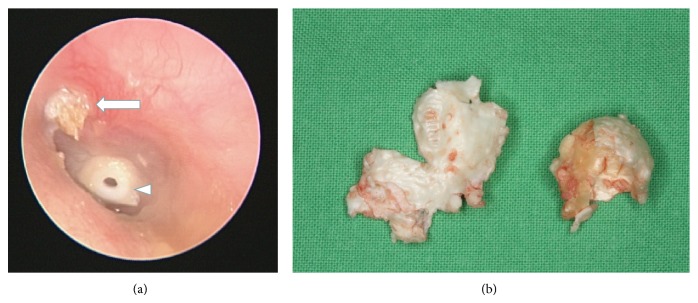
(a) Acquired cholesteatoma of attic in the left ear (arrow). Accumulation of debris within an attic retraction pocket led to gradual expansion of cholesteatoma. Grommet insertion revealed poor ventilation function in the middle ear (arrow head). (b) Dissected friable cholesteatoma with a thin pearly-white greasy-looking wall containing pultaceous substance.

Cholesteatomas can be classified as one of two different types: congenital, which is specific to childhood, and acquired, which affects children as well as adults [9]. Congenital cholesteatoma is defined as a white mass that forms prior to birth behind an intact eardrum and has no history of otitis media or previous otologic procedures (Figure 1). Acquired cholesteatomas most commonly begin after birth with a retraction pocket in the eardrum, usually as a result of chronic middle ear disease (Figure 2) [1, 10]. Pediatric cholesteatoma is more frequently infectious, more aggressive, more proliferative, and associated with a less favorable prognosis [3, 11]. The primary characteristic differentiating pediatric acquired cholesteatoma from its adult counterpart lies in its clinical behavior.

The likelihood of bone erosion, the lack of effective, nonsurgical therapies, and the potentially fatal consequences of acquired cholesteatoma underline the need for a comprehensive investigation of this condition, particularly in children. This paper summarizes previous progress and contemporary advances in the treatment of acquired cholesteatoma. We also provide a comprehensive overview of related background issues, particularly with regard to pediatric cholesteatoma.

## 2. History and Etymology

The French anatomist Du Verney first reported a case of cholesteatoma-like symptoms in 1683 [12]. Nearly a century and a half later, in 1829, Cruveilhier described the pathologic features of what he referred to as pearly tumor (tumeur perlée), referring to its whitish pearly appearance (Figures 1 and 2) [13]. The term cholesteatoma (chole = cholesterol; steat = fat; oma = tumor) did not appear in the literature until 1838 [9], when Johannes Müller, a German anatomopathologist, coined the term to describe a tumor that appeared “greasy in nature” [3, 14]. Other denominations were also proposed, including “margaritoma” by Graigie in 1891 and “keratoma” by Schuknecht in 1974 [3]; however, they were never widely adopted. Today, the term “cholesteatoma” remains the dominant term in clinical practice [3, 15, 16]. Nonetheless, this is something of a misnomer because the lesion contains neither cholesterine nor fat, and it is not neoplastic in nature [3, 6, 15, 16]. A general misunderstanding of the disease entity is the reason that the term still holds much ambiguity among the general population.

## 3. Classification

There are two main types of cholesteatoma based on the disease pathogenesis: (1) congenital, which is specific to childhood, and (2) acquired, which affects children as well as adults [9]. Congenital cholesteatoma presents a nidus of trapped squamous epithelium behind an intact eardrum and has no history of otitis media or previous otologic surgery. Acquired cholesteatomas, localized exclusively in the middle ear, are further divided into primary acquired and secondary forms [2, 15]. Primary acquired cholesteatoma occurs as a retraction pocket in which desquamated keratin epithelium accumulates behind an apparently intact eardrum, usually in the region of the pars flaccida [2, 3, 17]. Secondary acquired cholesteatoma appears secondary to epithelial migration into the middle ear through a perforated eardrum, which is in turn caused by infection, trauma, or iatrogenesis [2, 15]. Tos proposed an otoscopic classification system based on the site of disease origination, in which cholesteatomas are divided into (1) attic, (2) pars tensa I (marginal disease), and (3) pars tensa II (central disease) [18]. In order to include cholesteatomas which occur behind an intact eardrum, Mills and Padgham added a fourth category, intact eardrum type, to this classification system [19]. Several other classification schemes have also been proposed based on a variety of criteria. In 1964, the Committee of Conservation of Hearing of the American Academy of Ophthalmology and Otolaryngology published a standard classification system to guide surgical treatment of chronic ear infections. Under this system, the type and location of cholesteatoma were included in the description of the gross disease present during surgery [20]. The rare disorder, petrous bone cholesteatoma, was classified by Sanna et al. into the following five categories: supralabyrinthine, infralabyrinthine, massive labyrinthine, infralabyrinthine-apical, and apical [21]. The above classifications were determined with the primary aim of comparing results between studies. Unfortunately, all previous attempts have failed to gain popular acceptance due to a lack of clinical significance. In addition to enabling an interstudy comparison of outcomes, a classification system should be able to identify patients who are at risk of cholesteatoma recidivism.

In 1984, Lien described a CAO classification system for cholesteatoma based on the extent of cholesteatoma (C), the degree of eardrum atelectasis (A), and the amount of ossicular erosion (O) [22]. That classification system revealed that most recurrent cases were in the advanced stage and appeared to have some prognostic benefits [23]. Unfortunately, a small sample size limited the validity of the classification system, such that further research using a larger data set was warranted.

In 1989, Tos and Lau proposed a system for the classification of cholesteatomas, which later became widely adopted [24]. This classification system includes three categories: (1) attic cholesteatoma, which develops from the pars flaccida (Shrapnell's membrane) and fills the Prussak space; (2) tensa retraction cholesteatoma, which develops from the retraction or adhesion of the entire pars tensa and involves the tympanic orifice of the Eustachian tube; and (3) sinus cholesteatomas, which develop from a posterosuperior retraction or perforation of the pars tensa and extend to the sinus tympani, posterior tympanum, and beyond [25–28]. Tos claimed that this classification system is useful for selecting the surgical procedure as well as for determining patient prognosis, wherein the lowest recurrence rate is associated with attic cholesteatomas, and the highest recurrence rate occurs in pars tensa retraction cholesteatomas. The prognostic value of the Tos classification system received support from Vartiainen and Nuutinen, who reported recurrence rates of 7.4% in attic cholesteatomas and 10.0% in pars tensa retraction cholesteatomas [29]. Unfortunately, the difficulties involved in defining the origin of advanced lesions tend to limit the clinical applicability of the Tos classification system and the effectiveness of associated prognoses.

In 1999, Saleh and Mills proposed an SOC system for the classification of cholesteatoma based on the site of cholesteatoma (S), ossicular damage (O), and preoperative complications (C) [30]. They reported that the SOC classification system reflects the severity of disease, and in that regard, it allows more meaningful comparisons to be made between published series.

In 2008, the Japan Otological Society (JOS) proposed a classification system for attic cholesteatoma based on the extent of the disease and associated complications [31]. The JOS later revised the classification system to fit pars tensa retraction cholesteatomas as well [32]. However, subsequent studies on the original [33] and revised [34] classification systems have failed to prove the prognostic validity of the methods.

In 2009, Telmesani et al. proposed an ATM system for the classification of cholesteatoma, including attic (A), tympanum (T), and mastoid (M), based on the extent of the disease [35]. In 2012, Belal et al. presented a TMC classification system of cholesteatoma based on the site of pathology in the tympanic cavity (T), its spread to the mastoid (M), and the presence of complications (C) [36]. A high correlation was observed between preoperative otoscopic and CT findings as well as intraoperative surgical findings for ATM and TMC classification systems. However, further study is required to prove that a correlation exists with prognostic predictions.

Despite these efforts, there remains no widely accepted standard for the classification of acquired cholesteatoma. To address this issue, a panel discussion was held during the 9th International Conference on Cholesteatoma and Ear Surgery in 2012 [37]. Analysis of responses among audience members revealed fundamental disagreements with regard to the progression of the disease and its severity at the individual level. Considerable work will be required to establish a classification system capable of achieving international consensus regarding the nomenclature of disease progression. Any such classification system will have to be relatively simple and easy to apply. It will also have to incorporate multiple clinical features, accommodate precise assessments of disease severity and progression, and provide a reliable mechanism for the prediction of prognosis.

## 4. Epidemiology

The annual incidence of acquired cholesteatoma ranges from approximately 9 to 12.6 cases per 100,000 adults and from 3 to 15 cases per 100,000 children [18, 38–41]. A male predominance of 1.4 : 1 in cholesteatoma incidence has been reported [16, 42]. Among children, a 72% preponderance of boys has been reported with no predilection for either the left or right side [43]. It should further be noted that the incidence of acquired cholesteatoma has declined in recent decades [44, 45], perhaps due to the widespread use of ventilation tubes [46].

Cholesteatoma prevalence has also been shown to vary according to race. Prevalence is highest among Caucasians, followed by Africans, whereas it is rarely observed in non-Indian Asians [9, 17]. The Inuit are a notable exception, who can exhibit cholesteatoma but with very low prevalence, due perhaps to a larger nasopharynx, which facilitates aeration of the middle ear, thereby preventing the subsequent formation of cholesteatoma sequelae [47]. The prevalence of cholesteatoma is higher in underdeveloped countries than in developed countries [41]; however, no difference in prevalence has been observed among various social groups [38]. Several families with multiple generations of affected individuals have recently been identified, which suggests there may be an underlying genetic propensity for cholesteatoma [48, 49].

In the pediatric population, cholesteatomas account for 10% of chronic otitis media cases [50]. Approximately 70–96% of cholesteatomas are acquired in nature [51, 52]. The mean age of children with acquired cholesteatoma has been estimated at 9.7 years (range: 6.4 to 13) [42]. It has also been estimated that 7–10% of affected children have simultaneous bilateral cholesteatomas or develop subsequent contralateral cholesteatomas during follow-up [53, 54]; both of these conditions are more common in girls [55].

It has been proposed that children with craniofacial syndromes are predisposed to develop cholesteatoma [56]. An estimated 0.9–5.9% of children with a cleft palate develop primary acquired cholesteatoma [56]. Among children with a cleft palate, it appears that grommet insertion does not alleviate the risk of developing cholesteatoma, with the incidence rate still ranging from 0 to 6.9% after insertion [57–65]. Indeed, children with a cleft palate face a likelihood of developing cholesteatoma that is 100–200 times higher than children who do not have a cleft palate [56, 60, 66].

## 5. Histopathology

**Figure 3:**
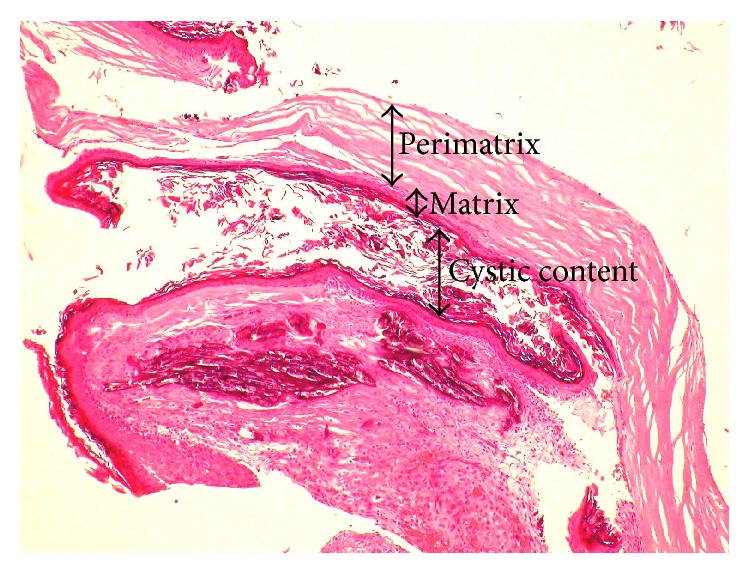
Histopathology of cholesteatoma showing a central mass of keratin (cystic content) surrounded by a thin layer of stratified squamous epithelium (matrix) and fibrous tissue with inflammatory infiltrate (perimatrix) (H and E, ×40).

At the macroscopic level, cholesteatoma presents as a whitish ovoid or round friable mass with a thin wall that contains pultaceous or a macerated substance. At the microscopic level, the lesion is divided into three layers: the cystic content, the matrix, and the perimatrix (Figure 3) [67]. The content of the cyst is the primary component of cholesteatoma, comprising fully differentiated anucleate keratin squames mixed with sebaceous material as well as purulent and/or necrotic matter. The matrix of cholesteatoma consists of hyperproliferative stratified squamous epithelium. As in the skin, the cholesteatoma epithelium comprises a basal layer (stratum germinativum), a spinal layer (Malpighian), a granular layer, and a lucid layer. The outermost layer is the perimatrix (lamina propria), an inflamed subepithelial connective tissue (granulation tissue) containing collagen fiber, fibrocytes, and inflammatory cells such as lymphocytes, histiocytes, plasma cells, and neutrophil leucocytes [17, 67, 68]. Pediatric cholesteatoma contains a greater proportion of cellular perimatrix than that found in adult cholesteatoma. Conversely, adult cholesteatoma contains a greater proportion of fibrotic perimatrix than does pediatric cholesteatoma. This may be an indication that pediatric cholesteatoma is more invasive and less reparative [69].

## 6. Pathogenesis

Most of the mechanisms that have been proposed to explain the pathogenesis of acquired cholesteatoma can be divided into four categories: (1) invagination theory (retraction pocket theory), (2) the theory of epithelial invasion or migration (immigration theory), (3) the theory of squamous metaplasia, and (4) basal cell hyperplasia theory (papillary ingrowth theory) [70]. Invagination theory is based on the assumption that the precursors to cholesteatoma are retraction pockets of the pars flaccida, which are caused by negative pressure in the middle ear [70, 71]. The subsequent accumulation of desquamated keratin in a deepening retraction pocket leads to the formation of cholesteatoma. Immigration theory contradicts the theory of invagination by assuming that eardrum perforations act as a precursor to cholesteatoma. The squamous epithelium of the eardrum then invades or migrates into the middle ear through a traumatic or iatrogenic defect in the tympanic membrane, leading to the formation of a cholesteatoma [72]. Conversely, squamous metaplasia theory states that middle ear mucosa can metaplastically transform into keratinizing epithelium, which subsequently leads to the formation of cholesteatomas [5, 70]. An enlarged cholesteatoma may then lead to tympanic membrane lysis and perforation, resulting in a condition with an appearance typical of acquired cholesteatoma. According to basal cell hyperplasia theory, keratin-filled microcysts, buds, or pseudopods formed in the basal layer of the pars flaccida epithelium invade the subepithelial tissue of Prussak's space, resulting in the formation of cholesteatoma [70, 73, 74].

A number of otologists believe that the mechanism underlying the pathogenesis of acquired cholesteatoma is a complex hybrid process involving all four of these theories, given that no single theory can fully account for uncoordinated hyperproliferation, invasion, migration, altered differentiation, aggressiveness, and recidivism [16, 70, 71]. Recent advances in technology have enabled researchers to investigate the etiopathogenesis of acquired cholesteatoma at the molecular level from the perspectives of immunohistochemistry and genetics.

### 6.1. Immunohistochemistry of Cholesteatoma

**Figure 4:**
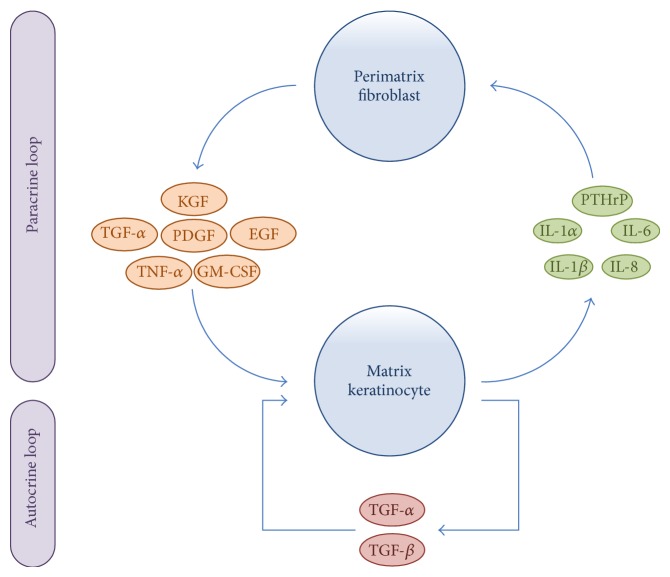
Schematic representation of the paracrine and autocrine interactions between matrix keratinocytes and perimatrix fibroblasts. Keratinocytes release proinflammatory cytokines (e.g., IL-1*α*, IL-1*β*, IL-6, PTHrP, and IL-8), which subsequently induce fibroblasts to secrete several cytokines (e.g., KGF, GM-CSF, EGF, TNF-*α*, PDGF, and TGF-*α*). These fibroblast-derived cytokines in turn induce the differentiation, proliferation, and migration of matrix keratinocytes. In addition, the TGF-*α* and TGF-*β* are upregulated in an autocrine loop, regulating keratinocyte proliferation and differentiation. EGF: epidermal growth factor; GM-CSF: granulocyte-macrophage colony stimulating factor; IL: interleukin; KGF: keratinocytes growth factor; PDGF: platelet-derived growth factor; PTHrP: parathyroid-hormone-related protein; TGF: transforming growth factor; TNF-*α*: tumor necrosis factor alpha.

Recent advances in immunohistochemical analysis have revealed an association between the progression of cholesteatoma and excessive host immune response to inflammation in the form of paracrine and autocrine secretions [70, 75–83]. As shown in Figure 4, paracrine and autocrine interactions between matrix keratinocytes and perimatrix fibroblasts regulate homeostasis and tissue regeneration within cholesteatomas [70]. In addition, inflammatory cell populations (e.g., monocytes, macrophages, and infiltrating leukocytes) in the matrix and perimatrix release a variety of angiogenic growth factors (e.g., vascular endothelial growth factor, epidermal growth factor, platelet-derived growth factor, interleukin-8, and cyclooxygenase 2) [84, 85]. These angiogenic factors subsequently promote angiogenesis, which paves the way for sustained migration of keratinocytes into the middle ear cavity through the provision of a new vascular network. Collectively, the recruitment of inflammatory cell populations in the matrix and perimatrix and their associated angiogenic growth factors are an important force driving the proliferation and aggressiveness of cholesteatoma. In addition, bone resorption is a mechanism that may explain the increase in osteolysis associated with acquired cholesteatoma. Following recruitment, bone marrow mononuclear cells are multinucleated to form osteoclasts [16]. Several upregulated cytokines in cholesteatoma have been shown to promote inflammatory bone resorption, including interleukin-1, interleukin-6, interleukin-17, interferon-beta, and parathyroid-hormone-related proteins [16, 86, 87]. Recent studies have revealed that the receptor activator of nuclear factor kappa-B ligand (RANKL) and matrix-metalloproteinases (MMPs) play a pivotal role in the destruction of bony tissue by cholesteatomas [16, 83, 88]. Furthermore, degradation of the extracellular matrix has been shown to be associated with the upregulation of MMPs (e.g., MMP1, MMP9, MMP10, and MMP12) as well as downregulation of the tissue inhibitor metalloproteinases (TIMPs) [89].

In summary, host innate immune response is a double-edged sword. Appropriate host immune response could offer protection against infectious threats; however, excessive inflammatory immune response can lead to the uncontrolled growth and proliferation of cholesteatoma.

### 6.2. Genetics of Cholesteatoma

Recent studies have demonstrated a link between pathogenesis and potential genomic alterations in cholesteatoma. The upregulation and activation of epidermal growth factor receptor (EGFR) and its ligand, transforming growth factor alpha (TGF-*α*), have been observed in several tumor types [90–92]. Overexpression of EGFR and TGF-*α* has also been detected in cholesteatomas, indicating that the dysregulation of these genes may be associated with the initiation and progression of cholesteatomas [93, 94]. Finally, alterations in the expression of protooncogenes (e.g., c-myc and c-jun) [95–99], upregulation of gap junction beta-2 (GJB2, also known as connexin 26) [89, 100, 101], and the downregulation of several tumor suppressor genes (e.g., p53, p27, CDH18, 19 and ID4, PAX3, LAMC2, and TRAF2B) [70, 76, 83, 89, 102] have been shown to contribute to the multifactorial pathogenesis of cholesteatoma.

Based on evidence collected thus far, cholesteatomas clearly exhibit clinical features similar to those observed in neoplasms, indicating that the dysregulation of cell growth control may involve internal genomic alterations. Nevertheless, further research will be required to reveal the precise underlying genomic mechanisms associated with the formation of cholesteatoma.

## 7. Clinical Presentations and Complications

Cholesteatomas often exist in a nonaggressive state, remaining undetected for years before potentially dangerous presentations manifest [103]. Indeed, the growth of cholesteatomas often progress undiscovered (i.e., untreated) until they have become dangerously large and threaten to invade intratemporal structures. This can lead to a number of intra- and extracranial complications [7, 104–110].

Many patients who suffer from cholesteatoma describe a frequently recurring and foul-smelling otorrhea, which is characterized as a scant but purulent discharge. Hearing loss can be progressive conductive or sensorineural. Conductive hearing loss is due to the impaired movement of ossicles, and further damage to the cochlea can cause irreparable sensorineural hearing loss, occasionally complicated by tinnitus. Destruction of the bone which overlies the semicircular canals (particularly the horizontal canal) can trigger vertigo or balance dysfunction [45]. In addition, violation of the facial nerve canal often manifests as temporary or even permanent facial paralysis. Prior to the advent of antibiotics, cholesteatomas were commonly subject to secondary infections [107]. Aerobic bacteria most commonly responsible for this include* Pseudomonas aeruginosa*,* Staphylococcus aureus,* and various* Proteus* species, whereas the most common anaerobic organisms associated with infection are* Bacteroides* and* Peptococcus*/*Peptostreptococcus* [39, 111]. Since the advent of antibiotic therapy, the incidence of secondary infections has fallen dramatically; however, clinicians must still be wary of infectious complications, due to the fact that cholesteatoma usually presents without symptoms of acute or chronic infection. In many cases, complications are not identified until they have progressed to advanced stages. A failure to control infection can lead to fatal complications, such as meningitis, brain abscesses, epidural abscesses, septic cavernous sinus thrombosis, and acute mastoiditis with a subperiosteal abscess [104, 107, 110].

Otalgia, headache, vomiting, and fever are nontypical presentations of cholesteatoma; however, their occurrence indicates the possibility of impending intratemporal or intracranial complications. Immediate assessment and treatment are required to prevent fatal consequences. Particular attention must be paid to the status of the ears of children, due to the common reluctance of pediatric patients to complain about symptoms associated with cholesteatoma, especially when presentations are subtle or unilateral [39]. Recurrent or persistent otorrhea over a period of two weeks should be treated as a possible warning sign of cholesteatoma, particularly when these symptoms persist despite treatment or in cases involving a suspicious hearing impairment in an ear that has previously been operated on [1].

## 8. Diagnosis

The potentially dangerous characteristics of cholesteatoma listed above may warrant particular attention by clinicians, even when symptoms appear to be mild. Early detection makes it possible to implement surgical measures that are less invasive than conventional treatments and can help guard against hearing loss, particularly in children.

### 8.1. Otoscopic Examination

**Figure 5:**
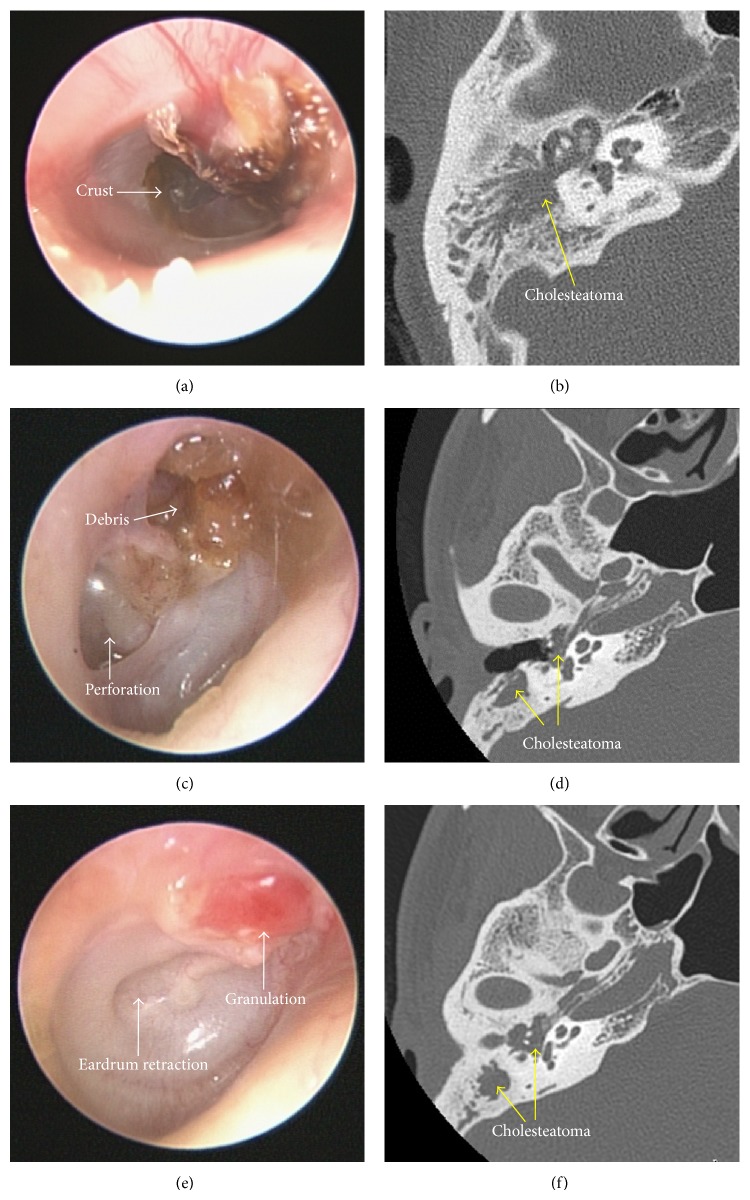
Cholesteatoma can be easily overlooked when hidden over the outer attic wall by various substances such as crust (a), debris (c), and granulation tissue (e). Complete removal of these substances may prevent misdiagnosis of cholesteatoma deep within the middle ear cleft ((b), (d), and (f)).

**Figure 6:**
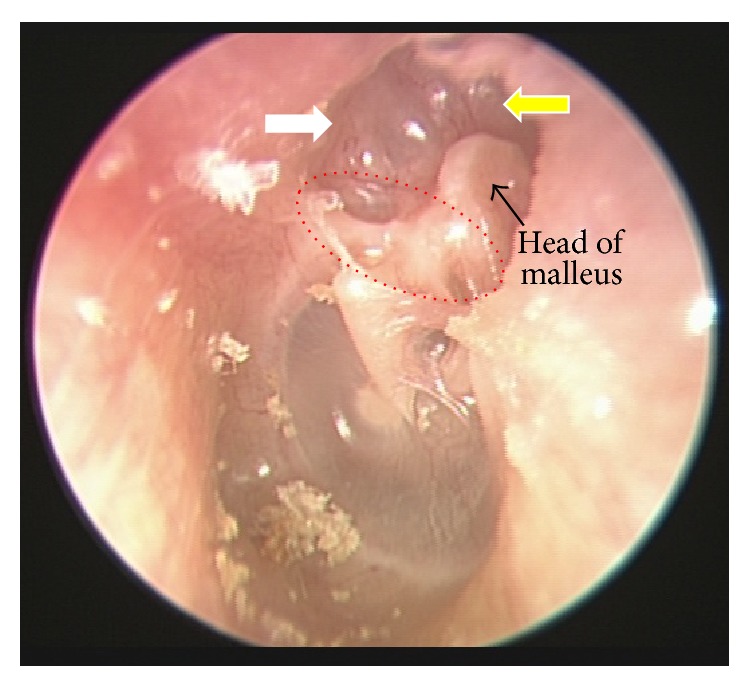
Attic retraction pocket in the left ear (white arrow) with atelectatic Prussak's space (red circle) and eroded scutum (yellow arrow).

Otoscopy, including pneumatic otoscopy, video-otoscopy, otomicroscopy, and video-telescopy, is the most direct and effective approach that can be used to inspect the eardrum. Ensuring sufficient brightness and illumination during an otoscopic examination is essential. The complete removal of all substances from the ear canal (e.g., cerumen, crust, debris, and granulation tissue) is also required to examine hidden areas and to avoid the misdiagnosis of cholesteatoma (Figure 5). The entire eardrum (extending to the outer edges) must be carefully examined and special attention should be paid to the attic and posterosuperior quadrant, which are the locations most commonly identified as the origin of acquired cholesteatoma (Figures 2 and 5) [112]. The aim of this examination is to identify any potentially cholesteatomatous lesions or their precursor, a retraction pocket (Figure 6).

During outpatient assessment, clinicians should be aware that the lesions associated with cholesteatomas and otitis externas may present similar appearances in otoscopic images [113]. Among patients with severe ear congestion or inflammation, cholesteatoma can be easily overlooked at the first presentation. If otitis externa is not resolved or tends to persist, then cholesteatoma should be suspected, and an otolaryngologic referral for detailed otologic examinations is warranted. Similarly, observations of suspicious cholesteatoma recidivism under otoscopic examination should prompt further image evaluation to determine whether a second look is warranted.

One important consideration that is commonly disregarded is the fact that most children with cholesteatomas also suffer from bilateral Eustachian tube dysfunction. Studies have shown that approximately 50% of these children present with abnormalities of the contralateral ear, and 7–10% of them eventually develop contralateral cholesteatomas [53, 54]. Thus, clinicians must be vigilant in their examinations and ensure continuous follow-up of the contralateral ear in order to detect even subtle otologic changes as early as possible. Difficult infants, uncooperative children, and cases involving suspicious cholesteatoma, as evidenced by otoscopic examination, should be referred to an otolaryngologist for further examination under anesthesia [39].

### 8.2. Computed Tomography (CT)

**Figure 7:**
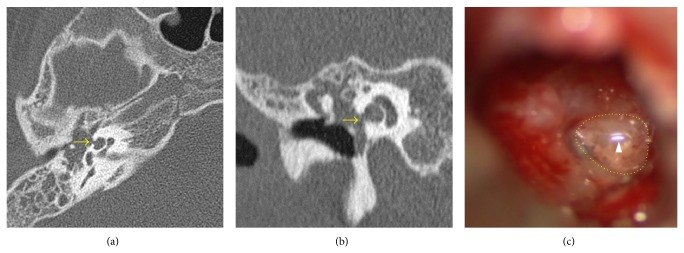
(a) Axial and (b) coronal CT scans of right temporal bone showing cholesteatoma with cochlear fistula (arrow). (c) Intraoperative findings confirmed the fistula with pulsatile fluid. The arrow head indicates light reflex in the fluid.

CT is the primary imaging modality employed in this area to determine the extent of the disease and assist in the planning of a surgical approach. Since its introduction in the early 1980s, high resolution CT (HRCT) has been the gold standard in the diagnostic imaging of cholesteatoma [114]. HRCT of the temporal bone is indispensable to otologists for surgical planning. Prior to surgery, HRCT scans should be examined repeatedly to identify the extent of disease, possible osseous destruction [115], anatomical abnormalities (e.g., middle ear hypoplasia, jugular bulb variations, bony dehiscence of the facial nerve and anomalies of its natural course, sclerotic or diploic mastoids, anterior sigmoid sinuses, and low-lying tegmens) [116], and other complications, such as tegmen dehiscence and labyrinthine fistulas (Figure 7) [117]. CT scans have a high negative predictive value in excluding cholesteatoma when there is no evidence of opacification in a well-aerated tympanomastoid cavity [118, 119].

It should be noted that similarities in the density of CT scans for cholesteatoma, granulation tissue, fibrous tissue, mucosal edema, and effusion greatly limit the ability of HRCT to distinguish among these disease entities [120]. CT scans are the preferable means to assess bone involvement, but this method is also somewhat limited in its ability to evaluate changes in soft tissue, such as changes associated with membranous labyrinthine or intracranial involvement [118]. In addition, the opacification frequently observed in CT scans following tympanomastoid surgery can detract from the reliability of postoperative recurrence or residual disease assessments [118, 119].

### 8.3. Magnetic Resonance Imaging (MRI)

Over the last decade, refinements in MRI algorithms, such as diffusion-weighted (DW) and delayed postgadolinium (DP) imaging sequences, have led to the development of alternative tools to detect cholesteatoma. Radiation-free imaging is of paramount importance to children due to their high radiosensitivity and longer life expectancy in which to develop radiation-induced cancers.

DW- or DP-MRI is a more accurate modality for the diagnosis of primary and relapsing cholesteatomas [118, 121, 122]. In the latest meta-analysis to evaluate the ability of DW-MRI to detect cholesteatoma, both overall sensitivity and specificity reached 94% [123]. Furthermore, compared with DW-MRI, DP-MRI is relatively time-consuming and unlikely to provide any additional information [118, 119]. This technique is therefore generally considered unnecessary [118, 124].

Two distinct sequences can be used for DW-MRI in the evaluation of cholesteatoma: echo-planar images (EPI) and non-echo-planar images (non-EPI). A recent systematic review of DW-MRI in the assessment of postoperative cholesteatomas suggested that non-EPI sequences are superior to EPI sequences in identifying recurrent or residual cholesteatoma. One important reason to apply non-EPI sequences is improved spatial resolution with a higher image matrix and fewer artifacts [118]. Non-EPI MRI has also demonstrated strong reliability in the detection of cholesteatomas smaller than 2-3 mm and has further shown good interobserver agreement [118, 123, 125–128]. Thus, non-EPI MRI is commonly used to complement preoperative CT imaging and has become a valuable tool to detect recurrent or residual cholesteatoma [129].

It has been estimated that advances in current imaging techniques could reduce the rate of second-look surgeries from 50 to 60% of cholesteatoma cases to 10% [130]. However, given the limitations of non-EPI MRI in assessing cholesteatoma pearls smaller than 2-3 mm in size [123, 128], whether this approach could completely replace second-look surgery remains doubtful [130, 131]. In the long run, it may prove safer to wait for suspicious small lesions to become evident or detectable by radiographic images or clinical examination [23]. Nevertheless, the necessity for a strict follow-up program to avoid delays in the detection of recidivism may cause great hardship for patients, particularly those who are unwilling or unable to make frequent visits to clinics. Under such circumstances, doctors and patients should weigh the benefits and risks of second-look procedures in order to permit more individualized decision-making.

Occasionally, surgical exploration may be considered a means of establishing a definitive diagnosis, particularly for patients who suffer from severe complications. Nonetheless, radiologists should be informed of any factors with the potential to cause false negatives with regard to DW-MRI signals, such as cerumen in the external auditory meatus, dental braces, an abscess cavity [132, 133], bone dust [134], or silastic sheets [119] identified during previous surgeries.

### 8.4. Ancillary Diagnostic Tools

Ancillary diagnostic techniques which can be used to evaluate cholesteatoma include audiometric and tympanometric testing. Audiometric testing is used to reveal deficits in conductive hearing. The manifestation of mixed hearing loss with a sensorineural component [17] may be associated with labyrinthitis [56]. If the ossicular chain is not involved, then hearing may be normal [135]. However, even in cases where the ossicles are damaged, hearing may appear deceptively unaffected or only mildly impaired due to the fact that the transmission of sound by the cholesteatoma can bridge the ossicular gap. This paradoxical phenomenon is referred to as “silent cholesteatoma,” “conductive cholesteatoma,” or “cholesteatoma hearer” [136, 137].

Tympanometric testing is usually performed in conjunction with audiometric testing to evaluate the condition of the middle ear. In addition to decreased compliance on the involved side, tympanometric findings may also indicate an eardrum perforation, which is less common in children than in adults. However, as with audiometric testing, tympanometric testing does not necessarily indicate the actual state of the middle ear. Unfortunately, no specific patterns associated with tympanometric changes are capable of providing a definitive diagnosis of cholesteatoma [138].

## 9. Nonsurgical Management

The application of antibiotic-steroid drops is required for patients with signs of acute infection (e.g., otorrhea) to reduce the occurrence of inflammation and granulation tissue [17]. Even before results of bacteriological culture analysis from ear discharge are available, empirical antibiotic therapy against possible infection by* Pseudomonas aeruginosa*,* Staphylococcus aureus*, or anaerobic bacteria is recommended [39, 111]. For this, the most appropriate antibiotics are fluoroquinolones (ciprofloxacin or levofloxacin) [111]. In addition, if patients suffer from advanced infection, oral or systemic antibiotics may be necessary.

However, the aforementioned medical treatment regimens are unable to provide a complete cure for acquired cholesteatoma; rather, these therapies are used to control preoperative infection or inflammation and to reduce the risk of postoperative complications [17, 139]. Despite voluminous research into the management of acquired cholesteatoma, a viable nonsurgical therapy has yet to be developed. It is hoped that current biomolecular advances in the understanding of cholesteatoma pathogenesis will prove beneficial in expanding the therapy spectrum and that future research will elucidate nonsurgical means to manage acquired cholesteatoma.

## 10. Surgical Treatment

### 10.1. Special Considerations for Children

Children with acquired cholesteatoma deserve special attention, given the more complex features associated with the pediatric form of the disease. For example, the underlying temporal bone of children is characterized by well-pneumatized air cells. Extensive mastoid pneumatization provides adequate space, which allows the disease to spread, exacerbating the aggressive behavior of pediatric cholesteatoma [46, 140]. Furthermore, the Eustachian tube is not fully developed in children. Specifically, it is shorter than that of adults; its opening to the nasopharynx is narrower; and it is positioned more horizontally [56]. The immature development of the Eustachian tube is believed to be closely associated with the rate of disease recidivism in children, which is 2- to 10 times higher than that of adults [3]. Thus, children are more likely to require revision surgery [3, 6, 141–145]. Finally, any delay in the diagnosis and/or treatment of cholesteatoma can have profound effects on language development, learning performance, and academic comprehension as a direct result of hearing loss [146, 147]. Early intervention increases the chance of preserving or reclaiming the hearing of patients; however, surgeons are typically unable to guarantee normal hearing after surgery.

The aforementioned characteristics specific to children pose a serious challenge to otologists who are forced to make decisions regarding surgical treatment of cholesteatoma. Given the disease complexity in the pediatric population, surgeons should provide comprehensive information about various surgical strategies to both parents and children in order to avoid misunderstanding and ensure fully informed consent.

### 10.2. Surgical Approaches: Canal Wall Up (CWU) and Canal Wall Down (CWD) Mastoidectomies

**Figure 8:**
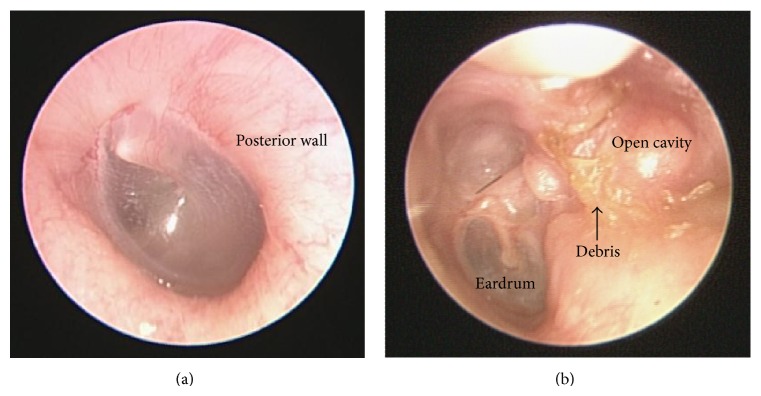
(a) Normal contour of the external ear canal in the left ear. (b) Traditional canal wall down mastoidectomy involves removing the posterior canal wall, which results in the formation of an open cavity. Regular ear cleaning is required to remove accumulated debris and control infection.

Numerous researchers have investigated different approaches to treat cholesteatoma; however, the primary treatment mode remains surgery. The main goal of surgery is to control disease, that is, to create a dry, trouble-free, and recurrence-free ear. Two types of surgical techniques are commonly applied: CWU and CWD mastoidectomies. CWU mastoidectomy necessitates the removal of all mastoid air cells while maintaining the integrity of contours in the ear canal [46, 148]. In contrast, CWD mastoidectomy involves removing the bony posterior canal wall to create a common cavity which combines the ear canal and mastoid (Figure 8) [149].

The pros and cons of CWU and CWD techniques have long been debated. The root of the controversy centers around whether the bony posterior canal wall should be preserved. In the CWU procedure, the maintenance of original normal contours, such as those of the external auditory canal, can help alleviate the disadvantages of creating a cavity in the CWD procedure, which include a lifelong aural toilet, water exposure limits, caloric stimulation vertigo when cold air or water enters the cavity, prolonged recovery, a cosmetically unpleasing appearance, and difficulties in filling hearing aids [46, 148, 150–153].

Conversely, proponents of the CWD procedure assert that the benefits are unlikely to outweigh the disadvantages of preserving the posterior bony canal wall. The compelling justification for foregoing the CWU procedure is the inability of the procedure to sufficiently expose critical areas of the middle ear cleft, despite the sacrifice of a substantial number of healthy mastoid air cells in order to gain surgical access to the cholesteatoma. Indeed, inadequate exposure is a major contributor to the higher incidence of residual disease in patients undergoing CWU, compared to those undergoing CWD, particularly when surgery is performed by less experienced surgeons [17, 148, 150, 154–156]. One recent meta-analysis on cholesteatoma recidivism rates revealed that patients undergoing CWU surgery are 2.87 times more likely to develop recidivistic disease than those undergoing CWD. Specifically, the recidivism rate ranges from 9 to 70% for CWU surgery and only between 5 and 17% for CWD techniques [150]. The higher probability of recidivistic disease necessitates taking a second look after 6 to 12 months [148] and occasionally a third or fourth look may also be required [17, 157]. Unfortunately, having to undergo repeated surgeries can impose intolerable suffering and economic burden on patients, especially those who are not covered by an insurance plan.

Various surgical approaches have been developed to address the drawbacks of CWU (high recidivism rates) and CWD (cavity problems) mastoidectomies. These modified methods include tailor-made tympanomastoidectomy with cartilage reconstruction [158], transcanal endoscopic management of cholesteatoma [159], and laser-assisted cholesteatoma surgery [160]. However, only minimal data related to outcomes is available, and long-term outcomes thus require further investigation [161].

### 10.3. Mastoid Obliteration

Mosher first introduced mastoid obliteration using a pedicled musculoperiosteal flap in 1911 to overcome problems associated with the postoperative mastoid cavity [162]. Since that time, numerous otologists have reported success with mastoid obliteration techniques, using alloplastic materials as well as various biogenic implants, such as fat, cartilage, bone pate, bone chips, ceramic powder, Ceravital, and hydroxyapatite [163–165].

Postsurgical problems involve the risk of a residual cholesteatoma remaining hidden within the obliterated cavity until it is too late for detection [166]. Unfortunately, this issue has not been well described in the literature, particularly for patients who suffer from a suppurative cholesteatomatous ear [163]. Kuo et al. recently published a report on the long-term (mean follow-up period of 12 years; range of 1–30 years) safety and sustained effectiveness of mastoid cartilage obliteration in 95 suppurative cholesteatomatous ears on 94 children (≤18 years). Their results showed comparable cumulative recidivism rates (including recurrent and residual cases) between patients that underwent mastoid cartilage obliteration and those that did not (*P* = 0.762, log-rank). Even when the authors excluded patients suffering from recurrent disease, the cumulative rates of residual cholesteatoma remained comparable between the two groups (*P* = 0.425, log-rank). This study provided evidence to support the long-term safety, feasibility, and effectiveness of mastoid cartilage obliteration. Nonetheless, further research is required to confirm these findings for different obliteration materials.

### 10.4. Hearing Interventions

The secondary role of cholesteatoma surgery is to either maximize hearing or restore serviceable hearing whenever possible. The management of hearing impairment depends on the type of hearing loss and the degree to which hearing can be restored. For instance, patients with minimal to no hearing loss may not require further hearing interventions. Furthermore, hearing aids may be a practical solution for patients with sensorineural hearing loss, and reconstitution of the sound transformation mechanism (i.e., ossicular chain) may be beneficial for patients with conductive hearing loss caused by the destruction of ossicles. Ossiculoplasty can be achieved through the placement of autologous grafts (e.g., bone or cartilage) [163] or a prosthetic device (e.g., partial or total ossicular replacement prosthesis) [167].

The choice of surgery may also significantly affect hearing outcomes. The hearing results of patients who receive CWD tend to be worse than those who undergo CWU due to impairment of resonance in the middle ear [168, 169]. Placement of a graft or prosthetic device in the middle ear may be also difficult for patients with CWD, given the altered middle ear space. Similarly, patients with CWD are limited in their choice of hearing aids, due to the enlarged ear cavity. Traditional hearing aids may be unsuitable in cases where the middle ear cannot be reconstructed or in cases of chronically draining ears. For these patients, bone anchored hearing aids (BAHA) present a viable option to improve hearing outcomes, as long as bone conduction remains intact [170].

### 10.5. Choice of Surgical Approaches

Several clinical considerations must be taken into account when choosing surgical approaches, as each technique has advantages and disadvantages with regard to surgical outcomes. Furthermore, surgeons have personal beliefs regarding specific techniques which are largely based on their own area of expertise. Practitioners should endeavor to be flexible when choosing surgical approaches [171]. Nonetheless, to a certain extent, the outcome of surgery is determined by the proficiency of the surgeon, regardless of surgical technique he or she chooses.

## 11. Minimum Follow-Up Time

Determining an appropriate duration for follow-up following cholesteatoma surgery is a challenging issue, and there is little hope of reaching consensus in the near future [23]. Nearly 90% of recurrent disease cases can be detected within 5 years after surgery; however, patients may be lured into a false sense of security and believe that they have been cured, despite the fact that a real threat remains [46]. In a recent long-term study (mean follow-up period: 15.4 years) on cholesteatoma, the mean duration prior to the detection of recidivism was found to be 10.4 years, and 71.4% of recidivism cases (5 in 7) were detected after more than 10 years. In fact, one case was discovered after 17.2 years. Clinical evidence from several long-term studies supports the late postoperative occurrence of recidivism, up to 15 years [172, 173] and 24 years [174]. Another long-term study revealed an increase in the rate of recidivism when the follow-up period was extended [157, 172–175]. To some extent, this may reflect the true course of the disease, which requires a time frame of sufficient duration to be identified. Thus, it is imperative that patients undergo periodic follow-up for as long as possible [23]. Nevertheless, it is important to bear in mind that the keys to minimizing recidivism are the complete eradication of disease and the consideration of recurrence risk factors, such as type of surgical treatment, the extent of disease, history of grommet insertion, and posterosuperior type of cholesteatoma [144, 163].

## 12. Knowledge Gaps in Cholesteatoma Management

Despite voluminous research on acquired cholesteatoma, knowledge gaps in the understanding of this disease remain. Result reporting is one of the major gaps that needs to be addressed. Recidivism rate (i.e., recurrent and residual rates) is a commonly used variable to report results. Many authors used the cumulative recidivism rate as the outcome of interest, which was calculated by summation of the recidivism rates obtained for each observation year [176]. Using the form of the standard rate calculation, however, the true risk of recidivism tends to be underestimated due to variations in length of postoperative follow-up period and rate of loss to follow-up [23, 176]. Recently, recidivism-free probability using Kaplan-Meier analysis has been proposed to ensure accurate and precise result reporting [23, 163, 176–178].

Other major gaps are related to (1) inconsistencies among reports with regard to the definition and classification of cholesteatoma; (2) discrepancies in the description of surgical techniques and modifications to these techniques, which can hinder the comparison of results; (3) the fact that, in most reports, follow-up periods are too short and dropout rates are too high to allow the extraction of useful data; (4) a failure on the part of many researchers to differentiate between pediatric and adult populations; and (5) the fact that different studies have used different methods to diagnose “residual cholesteatoma,” with some researchers using imaging, others using a routine second look, and still others waiting for pearls to become apparent. A number of studies have even failed to differentiate between residual and recurrent cholesteatomas. Some minor knowledge gaps are associated with the choice of CWU versus CWD techniques, treatment used for retraction pockets, and the application of monitoring techniques to reduce the risk of facial nerve injuries.

In conclusion, this paper provides a comprehensive summary of past and current developments in acquired cholesteatoma research and also describes a number of conceptual insights. First, the incidence of acquired cholesteatoma has decreased in recent decades. Second, the most recent and arguably the most important development has been the development of novel MRI sequences in the early 2000s. These sequences are capable of facilitating the detection of primary and residual lesions. Unfortunately, despite improved understanding, the management of this disorder has progressed very slowly, and surgery remains the predominant management technique. Exploring additional avenues of biomolecular research could expand the spectrum of therapeutic choices and will hopefully address the profound need for a feasible nonsurgical form of therapy to treat acquired cholesteatoma. Finally, maintaining follow-up for as long as possible is entirely warranted in order to detect late recidivism.
